# Comparison of SPAG11A gene expression in infertile men with grade 1 and 2 varicocele before and after treatment

**DOI:** 10.5935/1518-0557.20220060

**Published:** 2024

**Authors:** Sepide Amiri, Lida Gholizadeh, Azam Rasti, Maryam Peymani, Seyed Ali Mohammad Mirjalili, Seraj-Aldin Vahidi, Seyed Mehdi Kalantar

**Affiliations:** 1 Department of Tissue Engineering and Applied Cell Sciences, School of Advanced Technologies in Medicine, Shiraz University of Medical Sciences, Shiraz, Iran; 2 Department of Biology, Faculty of Basic Sciences, Islamic Azad University, Shahrekord Branch, Shahrekord, Iran; 3 Research and Clinical Center for Infertility, Yazd Reproductive Sciences Institute, Shahid Sadoughi University of Medical Sciences, Yazd, Iran; 4 Abortion Research Centre, Yazd Reproductive Sciences Institute, Shahid Sadoughi University of Medical Science, Yazd, Iran; 5 Department of Medical Genetics, School of Medicine, Tehran University of Medical Sciences, Tehran, Iran; 6 Department of Andorology, Yazd Reproduction Sciences Institute, Shahid Sadoughi University of Medical Sciences, Yazd, Iran

**Keywords:** varicocele, *SPAG11A*, epididymis, semen analysis, real time PCR (qRT-PCR)

## Abstract

**Objective:**

Sperm Associated Antigen 11A (*SPAG11A*) protein is a family of the epididymis-specific secretory proteins implicated in sperm maturation and function. Varicocele might cause pathophysiological difficulties in the testis and epididymis, with a harmful effect on the environment for spermatogenesis and sperm maturation. The aim of this study was to evaluate the expression level of the *SPAG11A* gene and sperm parameters in infertile men with grade 1 and 2 varicocele before and after treatment.

**Methods:**

Semen specimens were collected from 20 infertile men with varicocele pre-and post-treatment and 10 healthy volunteers. Semen analysis was conducted according to world health organization guidelines. Real time PCR (qRT-PCR) reaction was applied for determination of *SPAG11A* mRNA expression.

**Results:**

The results showed that there was a significant difference between the concentration and normal morphology between pre- and post-treatment groups and the controls. There were significant differences between pre-treatment and control groups in terms of progressive and non-progressive mobility. *SPAG11A* mRNA levels were significantly lower in the pre-treatment group than in healthy control subjects (*p*=0.007). There was no statistically significant difference in the expression of *SPAG11A* as well as semen parameters in the post-treatment group compared to the pre-treatment group.

**Conclusions:**

*SPAG11A* gene expression and semen parameters may be affected by varicocele. Whether varicocele treatment is an effective approach to reduce the adverse effect of this disease on *SPAG11A* expression and semen parameters needs further investigation.

## INTRODUCTION

Spermatozoa going out the testis undergo maturation and gain fertilizing ability and forward movement during their crossing through the epididymis, which provides the microenvironments for their maturation (Tian *et al*., 2012). Different proteins secreted into the epididymal lumen cause morphological and molecular changes in sperm maturation. Proteins of the *SPAG11A* family are known to be localized on the sperm surface (Sangeeta & Yenugu, 2020), also known as human epididymis 2 (HE2) in humans that bind to the head and neck regions of spermatozoa (Ribeiro *et al*., 2012). There are two *SPAG11* genes, known as *SPAG11A* and *SPAG11B* and in the defensin gene cluster on chromosome 8p23 in humans (Dorin & Barratt, 2014). In addition to their activities in male tract host defense, *SPAG11* isoforms and other b-defensins are also involved in sperm maturation and function by affecting sperm motility and zona-pellucida recognition (Zhou *et al*., 2013).

Cause of varicocele is determined by a dilated pampiniform plexus, the network of small veins responsible for venous drainage from the testicle and deep tissues of the hemiscrotum. The plexus is adjacent with the ipsilateral gonadal vein, which drains into the renal vein on the left and directly into the inferior vena cava on the right. The left renal vein is typically 8-10 cm length and has a higher hydrostatic pressure; this explains the difference in incidence between the left side and the right side; which when stiff and unilateral may be of concern for malignancy (Pastuszak & Wang, 2015). Based on physical examination, varicoceles are classified according to the Dubin and Amelar system as grade 1, 2 or 3 (Iosa & Lazzarini, 2013). Although varicoceles are associated with disruption of normal testicular function and spermatogenesis, the exact mechanisms that would ultimately lead to infertility are controversial (Jensen *et al*., 2017). Varicoceles often lead to alterations in the sperm count, motility, and morphology. Varicocelectomy is the most common treatment option of varicoceles (Garg & Kumar, 2016). Several investigators are evaluating antioxidants for the treatment of elevated levels of reactive oxygen species, this approach is still experimental (Dave *et al*., 2021). Some studies have established that varicocele treatment can result in a significant improvement in one or more semen parameters (Mahdavi *et al*., 2016).

Mechanisms by which varicocele affects fertility potential remain poorly understood. The early hypotheses involve hyperthermia, venous pressure, testicular blood flow, hormonal disbalance, toxic substances, and reactive oxygen species (Blumer *et al*., 2012). Elevations of epididymal temperature cause storage reduction and impaired spermiogenesis, causing changes in sperm parameters through increase of apoptosis (Shiraishi *et al*., 2012). Reasons such as mutations, polymorphisms, changes in gene expression, and epigenetic changes have all been linked with varicocele (Santana *et al*., 2017). Some investigators have demonstrated that varicocele treatment can alter the expression of genes important for semen quality (Oliveira *et al*., 2012; Amer *et al*., 2015). Since the coordinated expression of many genes is involved in the process of sperm maturation in the epididymis, deregulation of the expression of these genes may cause spermatogenesis disruption and infertility (Belleannée *et al.,* 2012).

The aim of this study was to evaluate sperm parameters and expression levels of the SPAG11A gene in infertile men suffering from varicocele grade 1-2 before and after treatment.

## MATERIALS AND METHODS

### Sample collection

This case-control study was conducted on 20 infertile men with a history of infertility for at least 1 year with their wives having a normal gynecological evaluation. with varicocele grade 1-2 at the age of 24 to 41 years (as case group) and 10 age-matched healthy infertile men with no sign of varicocele (as controls), referred to the Andrology Laboratory of the Yazd Research and Clinical Center for Infertility from July 2016 and August 2017. All the men with varicocele were treated and/or managed by surgery or medication (Folic acid and vitamin E400) depending on the grade of varicocele. Semen specimens were obtained from all the subjects. In the case of men with varicocele, two semen samples were provided, one before treatment and one 3 months after treatment. Of the 20 men with varicocele, only 10 men referred back for post-treatment analyzes. Men who did not return for post-treatment examinations were excluded from the final analysis. A questionnaire including detailed information, such as age, cigarette smoking, drug abuse, alcohol drinking, occupation, exposure to toxic substances, previous surgery, medicine consumption and abstinence time was fulfilled by all participants.

### Semen analysis

Semen samples were obtained by masturbation after 2 to 7 days of sexual abstinence. After liquefaction, conventional semen analysis was conducted in accordance with the WHO guidelines. Sperm concentration and motility were evaluated by a Makler counting chamber (Sefi Medical Industries, Haifa, Israel). Motility was expressed as a percentage of progressive, non-progressive and/or immotile sperm. Smears of raw semen were stained using the Diff-Quik method (Rapid sperm staining kit, Dayan ZistAzma, Iran) for assessment of sperm morphology. The remaining semen sample of each subject was centrifuged at 3000 g for 10 min to collect sperm pellets and plasma fractions. The sperm pellet was stored at –80°C until future analysis in connection with the SPAGA11 mRNA expression assessment.

### mRNA extraction and qRT-PCR

The expression of SPAGA11 mRNA was determined by quantitative real-time PCR (qRT-PCR) using the ABI (Applied Biosystems™ Step One™ Real-Time PCR System). The total RNA was extracted from sperm using the RNA extraction kit (MN, Macherey Nagel, Germany) following the manufacturer’s instructions. The concentration and purity of RNA were assessed by NanoDrop Technologies. First-strand cDNA was synthesized from 1 µg of total RNA using Thermo Scientific RevertAid First Strand cDNA Synthesis kit (*Thermo Fisher, USA*). The obtained cDNA was stored at –20°C until used. For gene expression analysis, each PCR reaction was carried out in a final volume of 20 µl, containing 12 µl of SYBR Real QPlus Master Mix Green high ROX™ (Ampliqon, Denmark), 2.5 µl cDNA, 1 µl (10 pmol) of forward and reverse primers, and 3.5µl ddH_2_O. To identify the optimal annealing temperature of the PCR primers, we first ran one annealing temperature gradient. The cycling conditions were as follows: one initial denaturation cycle at 95°C for 7 min, followed by 35 cycles of denaturation at 95°C for 1 min, annealing at 58°C for 1 min, and extension at 72°C for 1 min. The qPCR assays were performed in duplicate. The sequences of the PCR primers used are shown in Table 1. To verify the specificity of the amplification products, we performed melting curve analysis at the end of each cycle series. The β–actin gene was considered as a reference gene for normalization of relative expression of target genes and the ∆Cts were calculated by the difference between Ct of the target gene and Ct of reference gene. Relative expression was calculated using the 2-^ΔΔ^Ct method.

**Table 1:** Primer sequences used for the real-time polymerase chain reaction.

Gene	Primer sequence (5’-3’)	Amplicon length (bp)
**SPAG11A**	F: 5' -CCAAGGGGATGTTCCACTGG-3' R:5'-AGCGTTGTCTGTGCTGCTG-3'	110
**β-ACTIN**	F:5' -CGCGAGAAGATGACCCAGATCATG3' R: 5'-CACCCACACTGTGCCCATCTACGT-3'	155

### Ethical consideration

This study was approved by Ethics Committee of the Yazd University, Yazd, Iran (IR.SSU.RSI.REC.1394.34). The recruited patients gave their informed written consent.

### Statistical analysis

The GraphPad software (GraphPad PRISM V 5.04) was used for the data analysis. The analysis of variance test (ANOVA) was used to assess the SPAG11A expression levels and sperm parameters difference among the different groups studied.

## RESULTS

### Expression of mRNAs in sperm samples of studied groups

**Figure 1:**
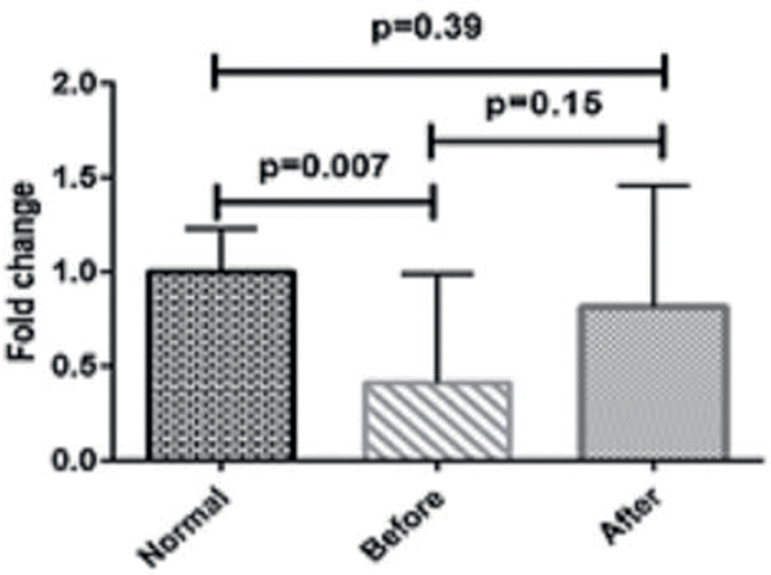
Comparison of the expression of SPAG11A mRNA in varicocele patients before and after treatment and normal subjects.

The mean age of infertile men with varicocele and normal subjects was 31.3±6.25 and 27.5±2.95, respectively (*p*=0.099). The expression levels of the *SPAG11A* gene were analyzed by quantitative RT-PCR in the infertile men with varicocele before and after treatment, as well as in healthy control subjects. The real-time PCR results showed that the expression levels of SPAG11A mRNA were slightly increased in post-treatment group compared with the pre-treatment one; however, it was not statistically significant (*p*=0.15) (Figure 1). As shown in Figure 1, SPAG11A mRNA levels are significantly lower in pre-treatment group than in healthy control subjects (*p*=0.007).

### Semen parameters

The results of semen parameters analysis are presented in Table 2. Sperm concentration was significantly lower in pre- and post-treatment groups in comparison with healthy controls (*p*=0.0001 and *p*=0.008, respectively) and it showed a statistically non-significant increase in post-treatment group compared to the pre-treatment one (*p*=0.09). There was a significant difference between the pre-treatment and control groups in progressive and non-progressive motility (*p*=0.001). Although, both the progressive and non-progressive motility showed an increasing trend in the post-treatment group when compared to the pre-treatment one, but it was not statistically significant (*p*=0.29 and *p*=0.24, respectively). Immotile sperm were significantly more common in the post-treatment group than among control subjects (*p*=0.04). Regarding the normal morphology, statistically significant differences were found between pre- and post-treatment groups compared with the control group (*p*=0.0001). Despite the increased rate of spermatozoa with normal morphology in the post-treatment group in comparison with the pre-treatment one, the result was not significant (*p*=0.054).

**Table 2:** Comparison of the conventional semen parameters between the groups.

Parameter	Before treatment	After treatment	Normal	*p*-values		
				Before *vs*. After	Before *vs*. Normal	After *vs*. Normal
Age	31.3±6.25		27.5±2.95		0.099	0.008
Volume (ml)	4.15±2.34	4.24±2.7	4.53±0.6	0.93	0.62	0.75
Concentration (mill)	47.2±30.13	78±46.55	130.4±31.33	0.09	0.001	0.008
None Progressive (%)	9.1±2.07	13.4±11.09	27.3±6.12	0.24	0.001	0.001
Progressive (%)	22.5±6.43	22.99±6.53	35±3.11	0.29	0.001	0.001
Immotile (%)	48.4±16.25	43.8±8.13	37.2±5.2	0.43	0.052	0.04
Normal Morphology (%)	1.7±1 15	3.6±2.67	6.13±2.06	0.054	0.001	0.001

Data presented as Mean±SD. ANOVA test, *p*<0.05 considered significant.

## DISCUSSION

In this study, we explored the expression of *SPAG11A* mRNA levels in varicocele patients pre- and post-treatment and compared them to those from healthy controls. To our knowledge, this is the first study evaluating the expression levels of *SPAG11A* gene in patients with varicocele. Our results indicated no statistically significant differences in the expression of *SPAG11A* mRNA before and after varicocele treatment compared with control subjects.

Spermatozoa are translationally and transcriptionally inactive cells. The conversion from immature to mature cells able of fertilizing an oocyte belong on post-translational modification of pre-existing proteins. These modifications happen through interactions together with proteins secreted by the epididymal epithelium as sperm traverse the epididymis (Gervasi & Visconti, 2017; Pujianto *et al.,* 2013). Most mammalian beta-defensin proteins, including members of the Sperm-Associated Antigen 11 family are some of the proteins that are detected predominantly in the epididymis (Sangeeta & Yenugu, 2019). *SPAG11* iso-forms not only play an important role in epididymal immunity but are also involved in sperm maturation by affecting sperm motility and zona-pellucida recognition (Ribeiro *et al*., 2016). The underlying mechanisms that regulate the expression of the epididymal genes are not well known but depend heavily on testicular androgens. Consistent with this finding, a study by Pujianto *et al*. (2013) revealed that *SPAG11A* is primarily regulated by circulating androgen. Thus, it seems that any changes in androgen levels may affect *SPAG11A* gene expression. Since, *SPAG11* isoforms play an important role in the maturation of sperm in human, variations in *SPAG11* can affect sperm quality (Dorin & Barratt, 2014).

In the present study, we found that *SPAG11A* mRNA levels were lower in patients with varicocele than in healthy controls, and these levels showed statistically significant differences between the pre-treatment group and controls (*p*=0.007). Referring to available evidence, decline observed in *SPAG11A* expression in varicocele men may be resulting from the secondary decline in androgen secretion by varicocele.

Machen *et al*. (2020) demonstrated that temperature sensing mechanisms that regulate the expression of epididymal genes are extremely sensitive and that artificially elevated scrotal temperature is likely to have negative effects on sperm maturation within the epididymis.

Therefore, another reason for the decline of *SPAG11A* expression in varicocele patients could be due to elevation of scrotal temperature caused by varicocele. In addition, our results showed that the expression levels of *SPAG11A* mRNA were slightly increased in post-treatment group compared with the pre-treatment one; however, it was not statistically significant (*p*=0.158). Another part of this study investigated the conventional semen parameters in varicocele men and control subjects. We compared pre- and post-treatment semen parameters such as semen volume, sperm concentration, motility, and morphology in all subjects. Our data on sperm analysis displayed no significant difference for semen parameters pre and post-treatment. However, there were statistically significant differences between the pre- and post-treatment groups compared with healthy controls in concentration and normal morphology of sperm (*p*<0.05). Regarding progressive and non-progressive motility, there was a significant difference between the pre-treatment and control groups (*p*<0.05). Although, sperm concentration, non-progressive motility and morphology were increased after treatment, but it was not statistically significant (*p*>0.05).

Several studies have shown disorders in sperm count, motility, and morphology in patients with varicocele and a significant recovery in these parameters following surgical correction. In addition, a recent metaanalysis found that varicocele has a significant effect on semen parameters including sperm count, motility, and morphology (Agarwal *et al*., 2016). The outcomes of a systematic review and metaanalysis demonstrate that varicocele was related with reduced sperm count, sperm motility, and sperm morphology although it had no effect on semen volume. Although varicocele is commonly considered to be the most common modifiable reason for male infertility, if varicocelectomy is an effective therapy for male factor infertility has been the subject of seriously discussion. Although the majority of studies indicate that varicocele repair improves sperm parameters, not all reports support this conclusion (Kupis *et al*., 2015; Ficarra *et al*., 2012).

There is much data that varicocelectomy is obviously related with significant improvements in sperm concentration, motility, and morphology (Kim, 2012; Shabana *et al*., 2015). A study by Al Bakri *et al.* (2012) showed that the average sperm count increased significantly after 3 to 6 months after varicocelectomy. Although, they have not been able to find any significant improvement with regards to semen volume, sperm count and total sperm motility after surgery.

Additionally, another study demonstrated that sperm motility, morphology and count 6 months after surgery was improved in patients with varicocele grade 1 and 2 (Asafu-Adjei *et al*., 2020). It has been shown that the repair of a higher grade varicocele results in a greater improvement in count and quality of spermatozoa compared with the repair of a lower grade varicocele (Hsiao *et al*., 2013; Samplaski *et al*., 2014).

In our study, we simply included the patients with varicocele grade 1 and 2 and assessed their semen parameters about 3 months after treatment. So, with respect to results from previous studies, it would be reasonable to suppose that the reason for not finding any statistically significant difference in semen parameters after varicocele repair in the present study may be the low grade of varicocele of our subjects, and that we failed to follow up our patients semen parameters beyond 3 months after treatment (Asafu-Adjei *et al*., 2020; Hsiao *et al*., 2013). There are several limitations in the present study. The main limitation of our study is its small sample size, which may have impacted our results. Another limitation of our study is that it came from a single center, which may not be representative of the general population of men with varicocele. In addition, we did not measure testosterone levels in varicocele patients before and after varicocele repair. The lack of follow-up semen analysis beyond 3 months after treatment is another limitation of this study.

## CONCLUSION

In summary, our findings showed no significant difference in the *SPAG11A* gene expression and conventional semen parameters before and after varicocele treatment. However, there was *a slight increase* in *SPAG11A* expression and sperm normal morphology following varicocele repair. Thus, it is reasonable to stress that varicocele may have negative effects on the expression of genes involved in sperm maturation, including the *SPAG11A,* and sperm parameters as well. There was no remarkable improvement with regards to *SPAG11A* expression and semen parameters after varicocele treatment, we cannot for certain propose varicocele repair as a good strategy to reduce adverse effect of this clinical problem. Further studies with a larger sample size are needed to support this claim.
